# Supplementary material for: The interactome of intact mitochondria by cross-linking mass spectrometry provides evidence for coexisting respiratory supercomplexes
Source: Mol Cell Proteomics. 2017 Dec 8;17(2):216–32. doi: 10.1074/mcp.RA117.000470 (PMC5795388; doi:10.1074/mcp.RA117.000470)
Supplement: Supplemental Data [file supp_RA117.000470_133922_0_supp_23978_fzffwf.zip › spectra_annotation/mito_spectra_annotation/61-1-4-1-3-1.pdf]

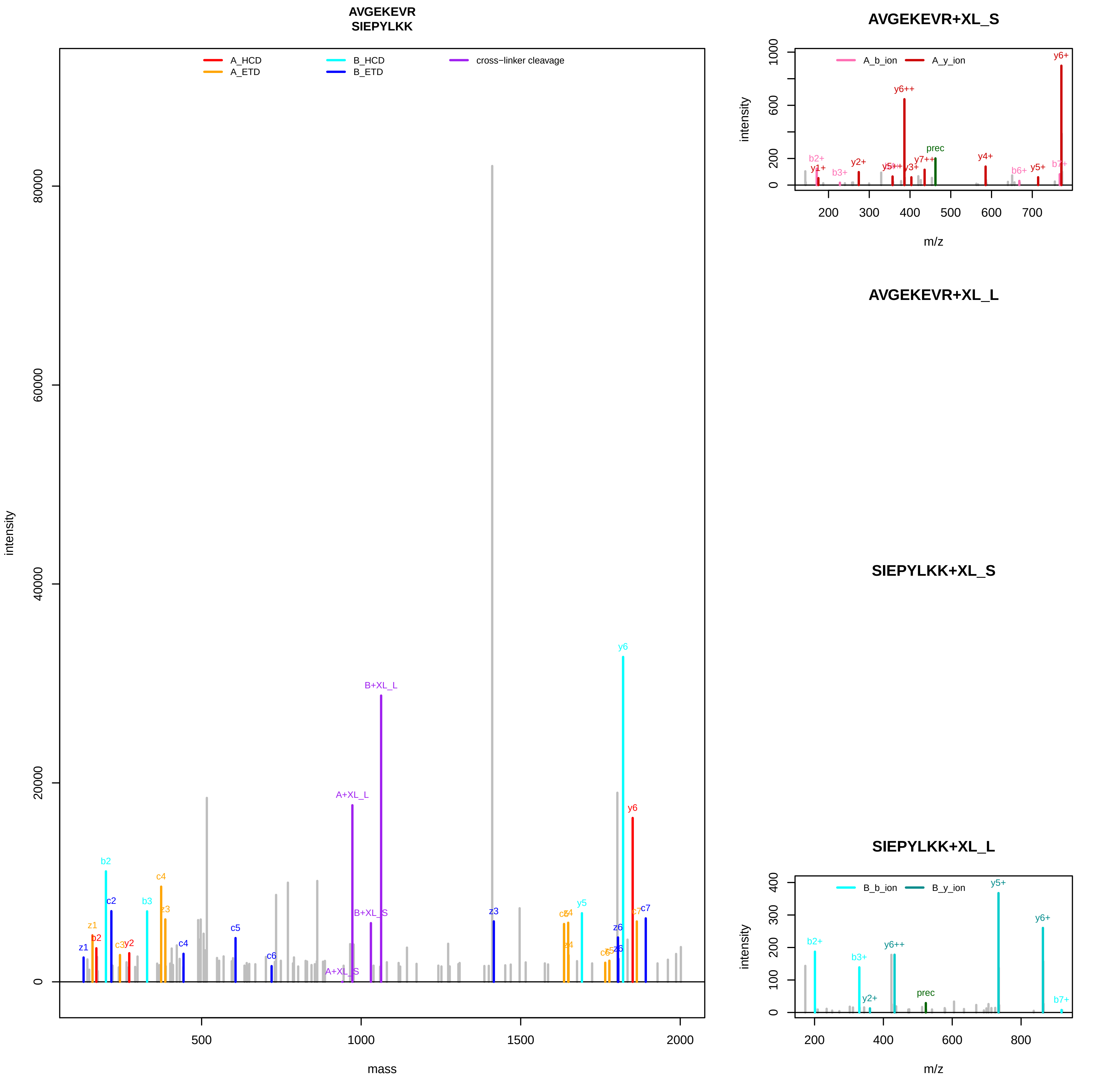

AVGEKEVR
SIEPYLKK
A_HCD
A_ETD
B_HCD
B_ETD
cross−linker cleavage
intensity
mass
B+XL_L
A+XL_L
B+XL_S
A+XL_S
y6
b2
c2
b3
c4
z3
c5
c6
c4
z1
b2
c3
y2
c5
z4
y5
z6
c7
AVGEKEVR+XL_S
A_b_ion
A_y_ion
y6+
y6++
prec
y4+
y7++
y2+
b2+
y1+
b3+
y3+
b6+
y5+
b7+
m/z
AVGEKEVR+XL_L
SIEPYLKK+XL_S
SIEPYLKK+XL_L
B_b_ion
B_y_ion
y5+
y6+
b2+
y6++
b3+
y2+
prec
b7+
m/z
